# An immune-relevant signature of nine genes as a prognostic biomarker in patients with gastric carcinoma

**DOI:** 10.1515/med-2020-0142

**Published:** 2020-09-03

**Authors:** Bing Wang, Yang Zhang

**Affiliations:** Department of Oncology, The Second Hospital of Dalian Medical University, No.467 Zhongshan Road, Shahekou District, Dalian, Liaoning, China

**Keywords:** gastric cancer, LASSO, immune, prognosis, signature

## Abstract

**Background:**

As one of the most common malignant tumors worldwide, the morbidity and mortality of gastric carcinoma (GC) are gradually increasing. The aim of this study was to construct a signature according to immune-relevant genes to predict the survival outcome of GC patients using The Cancer Genome Altas (TCGA).

**Methods:**

Univariate Cox regression analysis was used to assess the relationship between immune-relevant genes regarding the prognosis of patients with GC. The least absolute shrinkage and selection operator (LASSO) Cox regression model was used to select prognostic immune-relevant genes and to establish the signature for the prognostic evaluation of patients with GC. Multivariate Cox regression analysis and Kaplan–Meier survival analysis were used to assess the independent prognostic ability of the immune-relevant gene signature.

**Results:**

A total of 113 prognostic immune-relevant genes were identified using univariate Cox proportional hazards regression analysis. A signature of nine immune-relevant genes was constructed using the LASSO Cox regression. The GC samples were assigned to two groups (low- and high risk) according to the optimal cutoff value of the signature score. Compared with the patients in the high-risk group, patients in the low-risk group had a significantly better prognosis in the TCGA and GSE84437 cohorts (log-rank test *P* < 0.001). Multivariate Cox regression analysis demonstrated that the signature of nine immune-relevant genes might serve as an independent predictor of GC.

**Conclusions:**

Our results showed that the signature of nine immune-relevant genes may potentially serve as a prognostic prediction for patients with GC, which may contribute to the decision-making of personalized treatment for the patients.

## 1 Introduction

According to estimates from the GLOBOCAN 2018, approximately 18.1 million new cancer cases and 9.6 million deaths were reported worldwide [1]. Gastric carcinoma (GC) is one of the most common malignant tumors and the third leading cause of cancer-related deaths worldwide [2]. Gastric adenocarcinoma, which accounts for 90% of all GCs, is the most common histological type [3]. The early symptoms of GC are not obvious; the most common symptoms at diagnosis in patients with advanced stage are dyspepsia, weight loss, anorexia, and abdominal pain [4]. Despite significant development in the treatment of GC during the past decade, the prognosis of patients with GC remains unsatisfactory, with a 5-year relative survival rate of less than 40% [4,5]. Therefore, identifying effective potential diagnostic markers and therapeutic targets to combat GC and thereby contribute to improving survival outcomes in patients with GC is urgently needed.

Recent studies have demonstrated that the dysregulation of gene expression is strongly associated with tumor initiation, progression, and migration, highlighting the emerging roles of genes as potential diagnostic biomarkers and therapeutic targets in patients with various cancers, including GC [4,6,7,8]. Evidence shows that the immune system plays a vital role in cancer occurrence and development [9]. Several studies have described a potential association between gene expression and the development of GC [10,11,12]. However, there has been no signature to systematically assess immune-relevant genes and predict the prognosis of patients with GC.

In the present study, transcriptomic data and the corresponding clinical follow-up information were used to identify key immune-relevant genes with a significant prognostic value. We then constructed a survival model to predict the prognosis of patients using these key immune-relevant genes. The prognostic prediction value of the immune-relevant gene signature was also systematically verified.

## 2 Materials and methods

### 2.1 Patients and datasets

Clinical follow-up information and transcriptomic data (407 samples, Workflow Type: HTSeq-Counts) of GC samples were downloaded from The Cancer Genome Atlas (TCGA) (https://portal.gdc.cancer.gov/) database and were used as the training cohort. An independent dataset from the Gene Expression Omnibus (GEO: GSE84437) was included in our study as an external validation cohort with 433 patients, which sequencing platform used was the GPL6947 Illumina HumanHT-12 V3.0 expression beadchip. The gene expression with count values equal to zero in all samples was removed from further analysis. Patients without survival data or survival time of less than 30 days were excluded. The immune-relevant genes were downloaded from the Molecular Signatures Database v7.0 (MSigDB) (http://software.broadinstitute.org/gsea/msigdb/) [13]. Our study was approved by the Ethics Committee of the Second Hospital of Dalian Medical University.

### 2.2 Data processing

The transcriptomic data of GC samples were downloaded from the TAGA database. Based on the annotation in the GENCODE project (http://www.gencodegenes.org) and normalized using the variance-stabilizing transformation [14], we obtained 9,277 gene expression profiles. The mRNA microarray dataset GSE84437 was downloaded from the GEO database. Based on the annotation in the sequencing platform and pre-processing, we obtained 17,845 gene expression profiles. Finally, 525 common immune-relevant genes were identified from the intersection of the TCGA cohort, GSE84437 cohort, and MSigDB database and were used for further analysis.

Then, the univariate Cox regression analysis was used to examine the association between immune-relevant genes in relation to the prognosis of patients with GC. A value of *P* < 0.05 was considered statistically significant.

Subsequently, the least absolute shrinkage and selection operation (LASSO) Cox selection method was used to establish the survival-predicting model [15].

### 2.3 Functional enrichment analyses

To better understand the potential function of immune-relevant genes, the Gene Ontology (GO) and Kyoto Encyclopedia of Genes and Genomes (KEGG) analysis were used with the “clusterProfiler” R package [16]. The threshold of the false discovery rate (FDR) was set as less than 0.05.

### 2.4 Construction and assessment of the prognostic immune-relevant gene signature

The immune-relevant prognosis risk score was constructed using the LASSO Cox selection method at 10-fold cross-validation [17] using the “glmnet” R package [18]. The risk score for each patient was calculated, based on the immune-relevant gene expression weighted by its associated Cox regression coefficient. The prognostic immune-relevant gene signatures were shown as risk score = (expr_gene1_ × coefficient_gene1_) + (expr_gene2_ × coefficient_gene2_) + … + (expr_gene9_ × coefficient_gene9_). The “surv_cutpoint” function of the “survminer” R package was used to generate the optimal cutoff value of the signature score. The GC samples were assigned to two groups (low and high) according to the cutoff value of the signature score. After that, the area under the curve (AUC) was calculated to validate the predictive ability of the immune-relevant risk signature, using the “survivalROC” R package [19]. The “survdiff” function of the “survival” R package was used to evaluate the significance of the survival difference between low- and high-risk groups [20]. The chi-square test was used to examine the association of the different clinical parameters between the two groups. Additionally, the overall survival (OS) was compared between the low-risk and high-risk groups using the Kaplan–Meier survival curve. Univariate and multivariate Cox regression analyses were used to evaluate whether the signature was independent of other clinical parameters. To further explore the potential biological effects of genes in the signature, the immune gene signature network was constructed with the Metascape (http://metascape.org/) online tool. We also used the Oncomine (https://www.oncomine.org/resource/main.html) and TIMER databases (https://cistrome.shinyapps.io/timer/) to check the expression level of genes in the signature in patients with GC and normal gastric tissues. The Human Protein Atlas (http://www.proteinatals.org) was used for immunohistochemistry validation to examine the protein levels of genes in the signature. All analyses were conducted in the R version 3.6.1 and SPSS version 25.0. A value of *P* < 0.05 was considered statistically significant.

## 3 Results

### 3.1 Construction and evaluation of the signature of nine immune-relevant genes

A total of 39 prognostic immune-relevant genes were identified using univariate Cox proportional hazards regression analysis. We then used the LASSO Cox regression model with 10-fold cross-validation to select genes with the best prognostic value. The nine immune-relevant genes were identified, and the risk score was calculated, based on their expression level and associated Cox regression coefficient. The risk score was calculated with the following equation:

\begin{array}{l}\text{Risk}\hspace{.5em}\text{score}\hspace{.25em}=\hspace{.25em}({\text{expr}}_{\text{CLEC}4\text{M}}\times 0.551)+({\text{expr}}_{\text{NOX}4}\times 0.242)\\ \hspace{1em}+({\text{expr}}_{\text{APOD}}\times 0.113)+({\text{expr}}_{\text{PROC}}\times 0.329)\\ \hspace{1em}+({\text{expr}}_{\text{VTN}}\times 0.073)+({\text{expr}}_{\text{GFAP}}\times 0.064)\\ \hspace{1em}+({\text{expr}}_{\text{CMTM}3}\times \mathrm{0.0.081})+({\text{expr}}_{\text{EGF}}\times 0.213)\\ \hspace{1em}+({\text{expr}}_{\text{CRHR}1}\times 0.583)\end{array}

**Figure 1:**
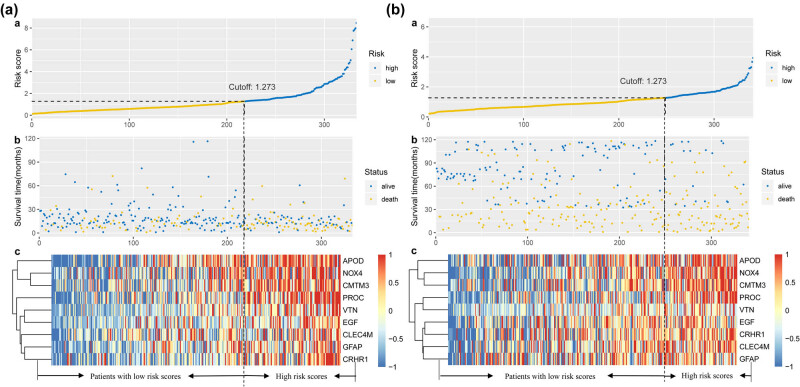
The prognostic signature in gastric cancer. (a) TCGA cohort: (a) risk score of each colon cancer: the risk score increased from yellow to blue; (b) survival time of each colon cancer: blue and yellow scatter represent alive and dead, respectively; (c) heatmap of the signature of nine immune-relevant genes. (b) GSE84437 cohort: (a) risk score of each gastric cancer: the risk score increased from yellow to blue; (b) survival time of each colon cancer: blue and yellow scatter represent alive and dead, respectively; (c) heatmap of the signature of nine immune-relevant genes.

**Figure 2:**
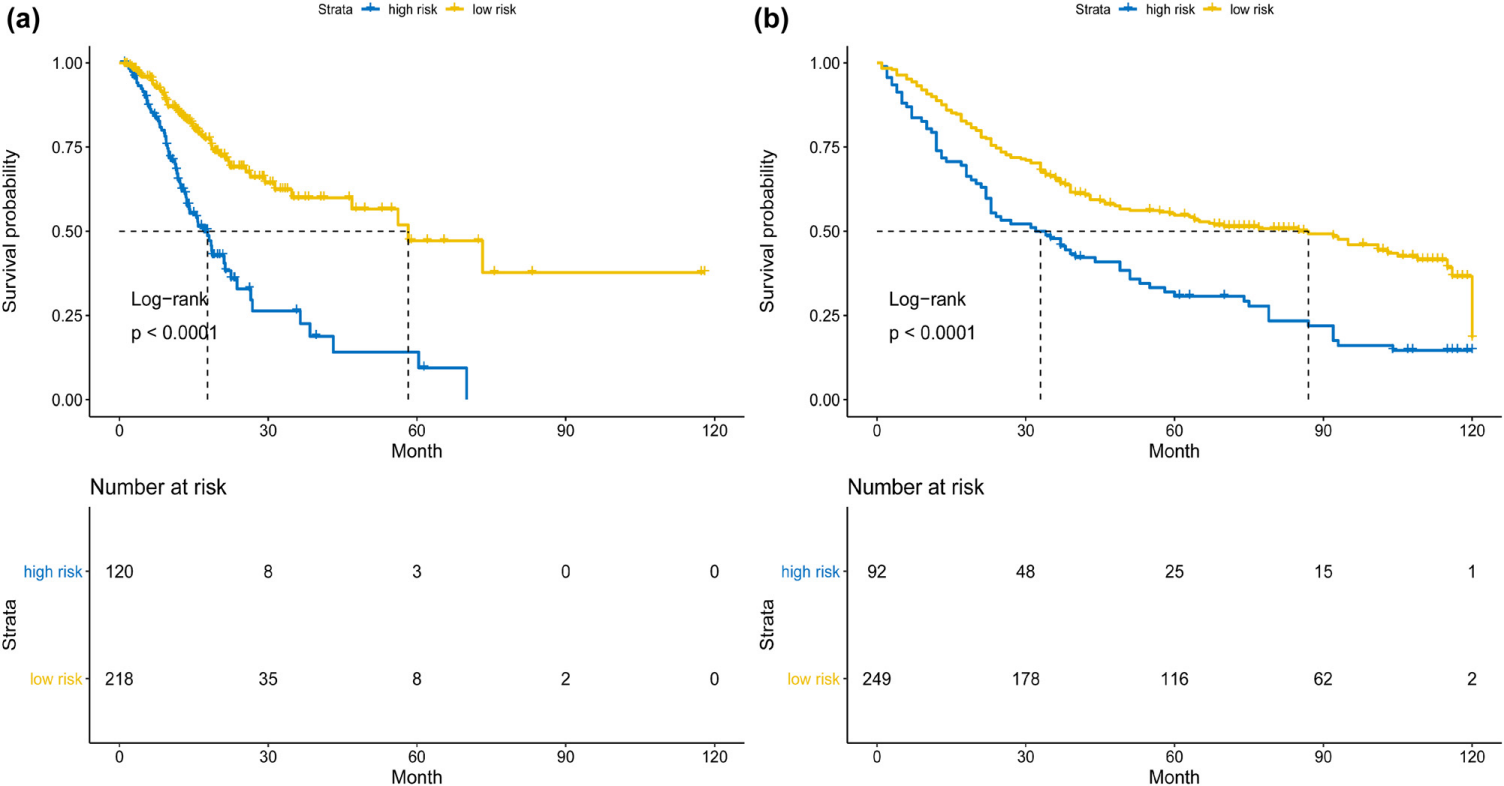
Kaplan–Meier curves of OS stratified by the nine immune-relevant genes’ signature score in high- and low risk for patients. (a) TCGA cohort and (b) GSE84437 cohort.

**Figure 3:**
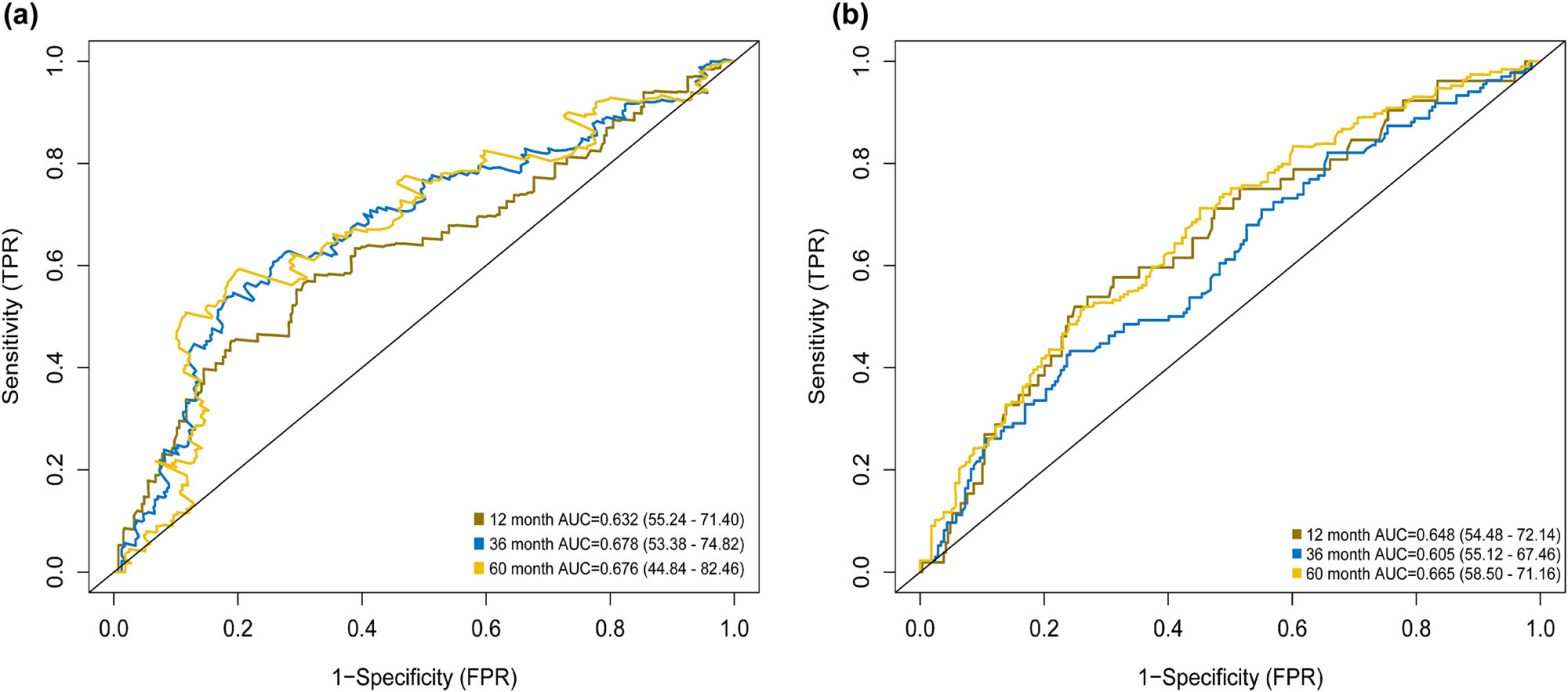
Time-dependent ROC curves of OS for the nine immune-relevant genes’ signature score. (a) TCGA cohort and (b) GSE84437 cohort at 1-, 3-, and 5 years.

Based on the optimal cutoff value of 1.273 for the risk score, samples were further divided into low- and high-risk groups (Figure 1a). The Kaplan–Meier log-rank test showed that high-risk patients had worse OS, compared with low-risk patients in the training cohort (Figure 2a, *P* < 0.001). In the time-dependent ROC curve analysis, the AUCs for 1-year, 3-year, and 5-year OS were 0.632, 0.678, and 0.676, respectively (Figure 3a).

### 3.2 Validation of the immune-relevant gene signature of nine genes in the GSE84437 cohort

We further validated the predictive ability of the immune-relevant signature of nine genes in the GSE84437 cohort. Based on the optimal cutoff value of 1.273 for the risk score, patients were assigned to low- and high-risk groups (Figure 1b). The Kaplan–Meier log-rank test demonstrated that the OS of the low-risk patients had significant survival advantages, compared with that of high-risk patients in the validated cohort (Figure 2b, *P* < 0.001). The AUCs for 1-year, 3-year, and 5-year OS were 0.648, 0.605, and 0.665, respectively, (Figure 3b).

These results demonstrate the great applicability and stability of the immune-relevant gene signature for predicting the prognosis of patients with GC.

### 3.3 Correlation of the immune-relevant gene signature with clinical parameters

Based on the optimal cutoff value of the risk score, patients were assigned to low- and high-risk groups. The associations identified between the signature of the nine immune-relevant genes and the clinical parameters of GC cases are summarized in Table 1. To further confirm the clinical value of the signature of nine immune-relevant genes, patients in each cohort were classified into low- and high-risk groups based on the OS-related clinical features (age, sex, grade, American Joint Committee on Cancer stage, T stage, N stage, and M stage) to evaluate whether the immune-relevant gene signature remains a powerful predictive ability. The log-rank test suggested that the OS in patients with GC was significantly longer in the low-risk group than in the high-risk group (Figures S1 and S2).

**Table 1:** Correlation between the clinical features of GC and nine immune-relevant genes’ signature

Parameter	TCGA cohort (*n* = 338)			GSE84437 cohort (*n* = 341)		
	High risk	Low risk	*P*	High risk	Low risk	*P*
Age (years)			**0.008**			0.626
<65	63	82		57	147	
≥65	57	136		35	102	
Gender			0.483			0.929
Female	40	81		28	77	
Male	80	137		64	172	
AJCC stage			0.740			
I + II	51	102				
III + IV	60	111				
Grade			0.140			
G1 + G2	40	89				
G3	78	122				
T			0.318			0.083
T1–2	28	63		6	33	
T3–4	89	154		86	216	
N			0.155			0.079
N0	30	70		10	47	
N1–3	90	146		82	202	
M			0.874			
M0	108	195				
M1	12	23				

Statistic: between-groups comparison using the chi-square test.

Bold values indicate *P* < 0.05.

### 3.4 The immune-relevant nine-gene signature as an independent prognostic factor

**Figure 4:**
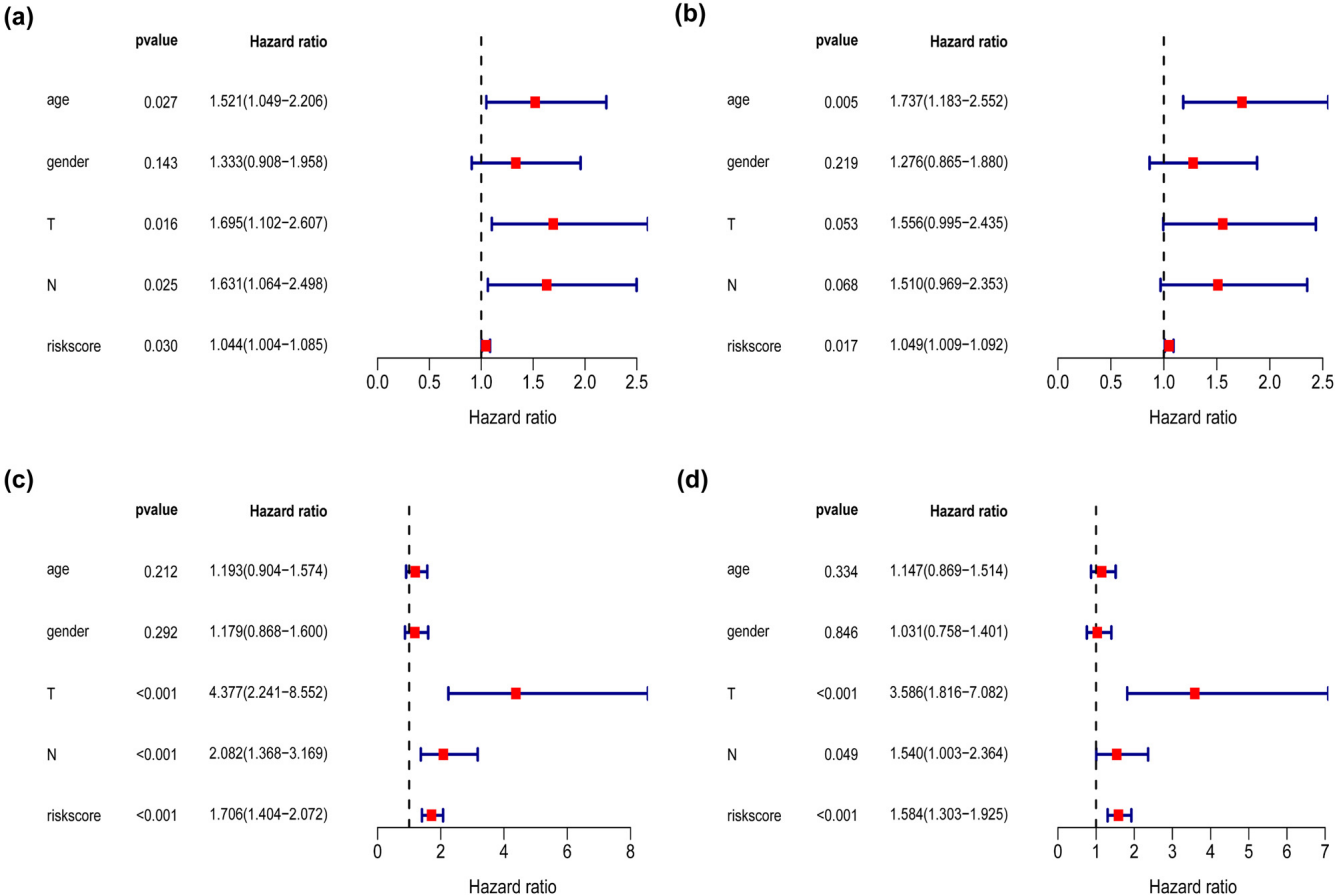
Univariate analysis and multivariate analysis of the correlation of the signature of nine immune-relevant genes with OS among GC patients in the TCGA cohort (a + b) and GSE84437 cohort (c + d).

Univariate and multivariate Cox regression analyses were used to evaluate and verify the independent prognostic factors in the TCGA and GSE84437 cohorts. The immune-relevant nine-gene signature was evaluated with several clinicopathological features (age, gender, grade, stage, T stage, N stage, and M stage) as covariables. Both in the TCGA and GSE84437 cohorts, the results of the univariate Cox analysis revealed that the signature of the nine immune-relevant genes was significantly associated with poor OS (TCGA cohort:hazard ratio (HR) = 1.044, *P* = 0.030; GSE84437 cohort: HR = 1.706, *P* < 0.001). Other clinicopathological parameters were also correlated with worse prognosis in patients with GC, including age, stage, T stage, M stage, and N stage (Table 2 and Figures 4a andc). Multivariate Cox analysis confirmed that the immune-relevant nine-gene signature was an independent risk factor for OS among patients with GC in both the test and validation cohorts (TCGA cohort: HR = 1.062, *P* = 0.006; GSE84437 cohort: HR = 1.584, *P* < 0.001). Other clinicopathological parameters were also correlated with worse prognosis in patients with GC, including age and M stage (Table 2 and Figure 4b and d).

**Table 2:** Univariate analysis and multivariate analysis of the correlation of nine immune-relevant genes’ signature with OS among GC patients

Parameter	Univariate analysis			Multivariate analysis		
	HR	95% CI	*P*	HR	95% CI	*P*
TCGA cohort						
Age	1.52	1.05–2.21	**0.026**	1.74	1.18–2.55	**0.005**
Gender	1.33	0.91–1.96	0.142	1.28	0.87–1.88	0.219
T	1.69	1.10–2.61	**0.016**	1.56	0.99–2.43	0.053
N	1.63	1.06–2.50	**0.025**	1.51	0.97–2.35	0.068
Risk score	1.04	1.01–1.08	**0.030**	1.05	1.01–1.09	0.016
GSE84437 cohort						
Age	1.19	0.90–1.57	0.212	1.15	0.87–1.51	0.334
Gender	1.18	0.87–1.60	0.292	1.03	0.76–1.40	0.845
T	4.38	2.24–8.55	**<0.001**	3.59	1.82–7.08	**<0.001**
N	2.08	1.37–3.17	**<0.001**	1.54	1.00–2.36	**0.048**
Risk score	1.71	1.40–2.07	**<0.001**	1.58	1.30–1.92	**<0.001**

Bold values indicate *P* < 0.05. HR, hazard ratio; CI, confidence interval.

### 3.5 Functional enrichment analyses

**Figure 5:**
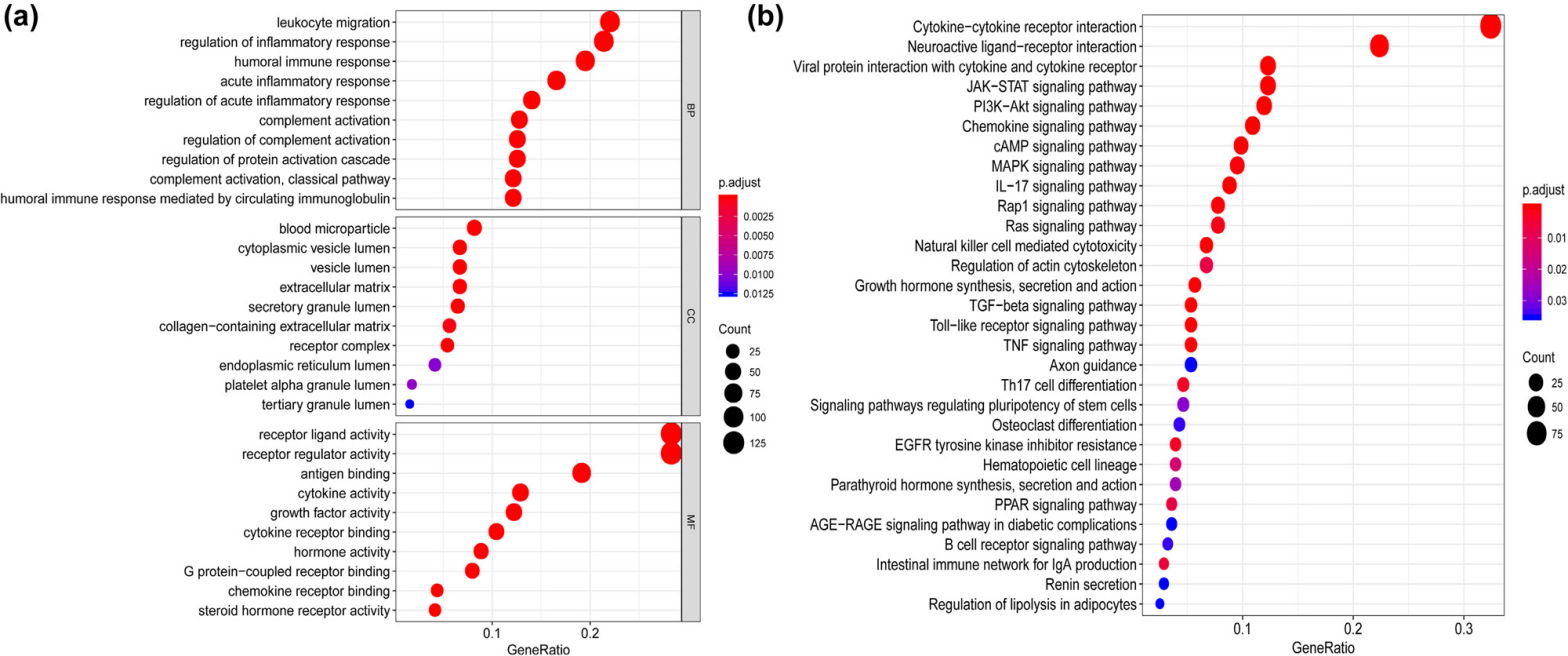
Gene functional enrichment of the immune-relevant signature. (a) The top ten most significant GO terms. (b) The top 30 most significant KEGG pathways.

**Figure 6:**
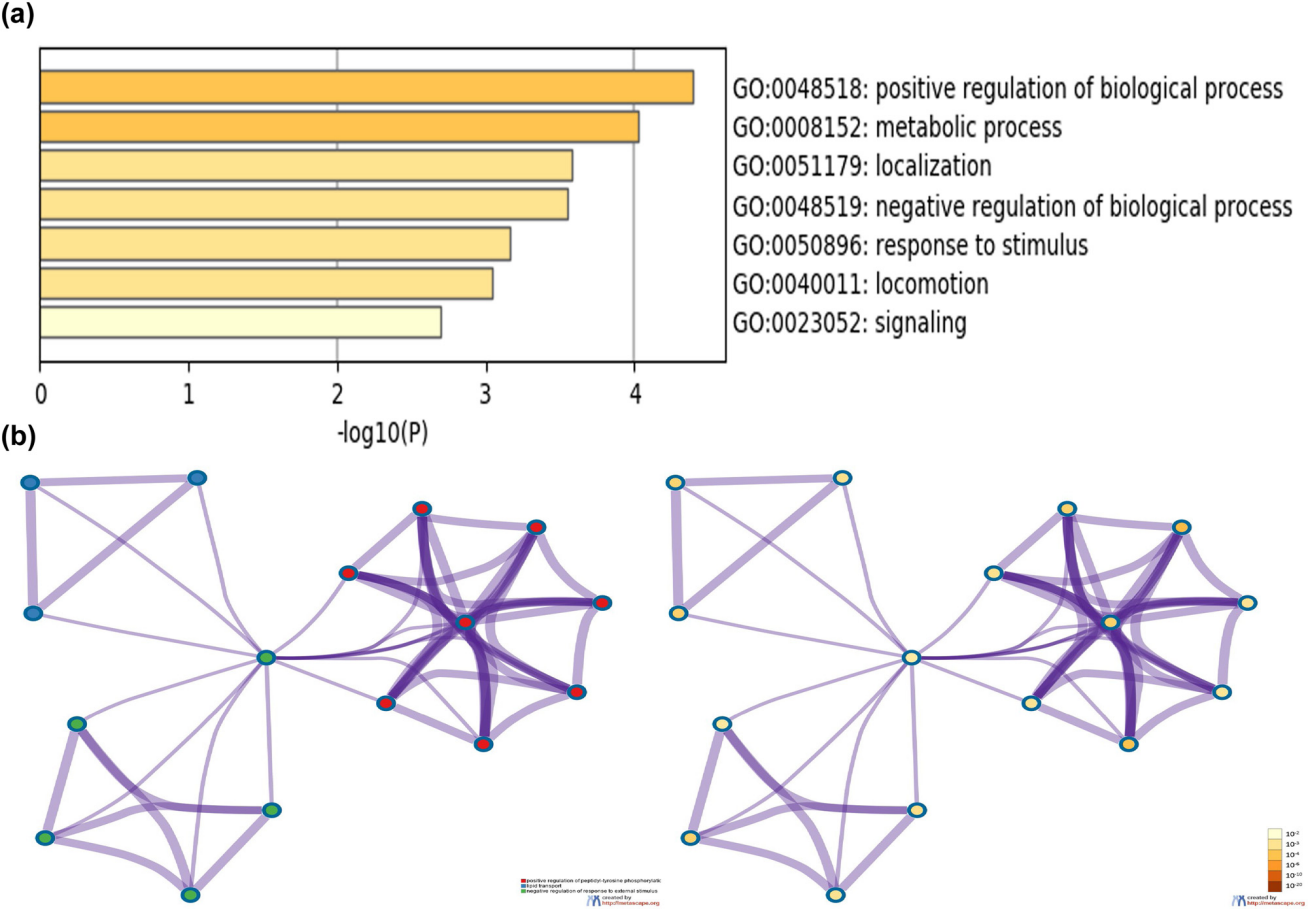
Functional and pathway enrichment analyses of the signature of nine immune-relevant genes. (a) GO terms and KEGG pathway are presented, and each band represents one enriched term or pathway colored according to the −log 10(*P*). (b) Network of the enriched terms and pathways. Nodes represent enriched terms or pathways, with node size indicating the number of genes of the immune gene signature involved in. Nodes sharing the same cluster are typically close to each other, and the thicker the edge displayed.

GO and KEGG enrichment analyses were performed to determine the biological functions of the immune-relevant genes. In the present study, a total of 1,115 GO terms and 50 KEGG pathways were identified with FDR < 0.05 as the statistical threshold. The top immune-relevant GO terms included inflammatory/immune response, leukocyte migration, complement activation, receptor regulator activity, cytokine/hormone activity, cytokine receptor binding, and chemokine receptor binding (Figure 5a). The top immune-relevant KEGG pathways included cytokine–cytokine receptor interaction, JAK-STAT, PI3K-Akt, chemokine, MAPK, TGF-beta, TNF, and B cell receptor signaling pathways (Figure 5b). To further explore the potential biological effects of the immune-relevant nine-gene signature, we constructed an immune gene signature network based on Metascape. The results revealed that the nine immune-relevant genes were enriched in the positive/negative regulation of biological processes, metabolic processes, localization, and response to stimulus (Figure 6).

### 3.6 External validation in online databases

**Figure 7:**
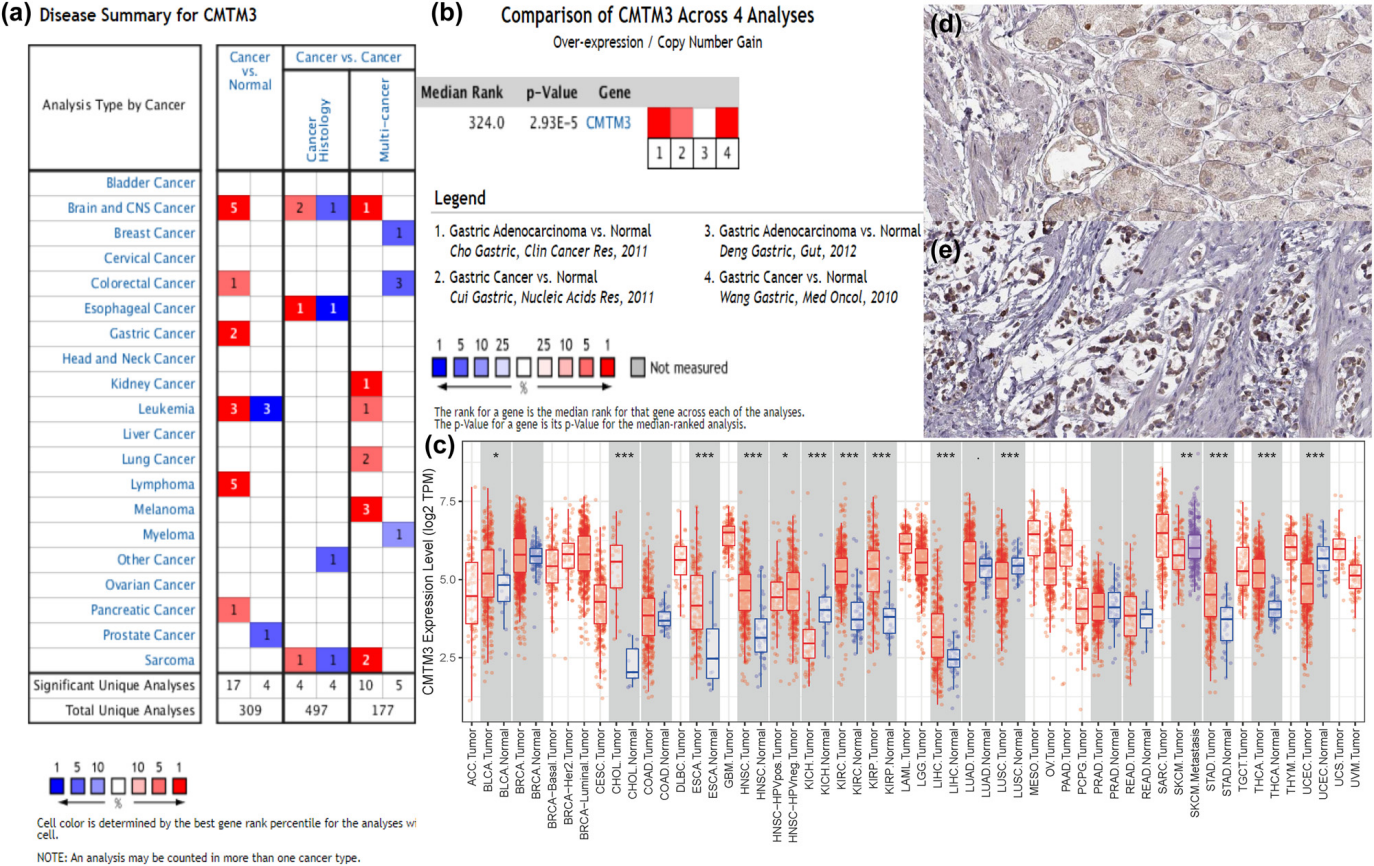
Expression analyses of CMTM3 by Oncomine, TIMER, and HPA databases. (a) The expression level of CMTM3 in different types of human tumors in the Oncomine database (https://www.oncomine.org/resource/main.html). (b) CMTM3 is significantly overexpressed in GC using four distinct gastric cancer datasets. (c) The expression level of CMTM3 in different types of human tumors in the TIMER database (https://cistrome.shinyapps.io/timer/). (d and e) The representative protein expression of CMTM3 in gastric cancer and normal gastric tissue. Data were from the Human Protein Atlas database (http://www.proteinatals.org).

Consistent with these results, CMTM3 was found to be significantly overexpressed in GC using four distinct gastric cancer datasets (Cho Gastric, Cui Gastric, Deng Gastric, and Wang Gastric) through pooled analyses in the Oncomine database (Figure 7a and b). Significant overexpression was also found in the TIMER database (Figure 7c). The representative protein expression of CMTM3 was also explored in the HPA database (Figure 7d and e).

## 4 Discussion

Despite significant development in the treatment of GC in the past 10 years, the patient prognosis remains poor. Most patients are diagnosed with an advanced stage; therefore, a simple surgical resection treatment may not achieve satisfactory results, and may need to be supplemented with radiotherapy or chemotherapy at the same time [4,5]. It is well known that various components of the immune system are involved in the occurrence and development of cancer [9]. Various studies have verified that GC is an immunogenic tumor and immunotherapy is strongly pursued targeting immune checkpoints [21,22,23]. Furthermore, the normalization of the immune microenvironment improves other antitumor therapies, including targeted therapy, radiotherapy, and chemotherapy [24]. Additionally, it is reported that several immune-relevant gene signatures are related to the sensitivity of a variety of chemotherapeutic drugs [8]. We therefore established a robust prognostic signature according to the immune-relevant gene using the TCGA-STAD datasets to predict patient survival outcomes.

As far as we know, this is the first report focusing on the association between prognostic immune-relevant gene signature and outcomes in patients with GC. This survival-predicting model consisted of nine immune-relevant genes with prognostic ability. The study demonstrated that our signature was significantly associated with OS in patients with GC in the TCGA and GSE84437 cohorts (TCGA cohort: *P* < 0.001; GSE84437 cohort: *P* < 0.001; Figure 2). These results demonstrate the great applicability and stability of the immune-relevant gene signature for predicting prognosis in patients with GC.

To examine the broad applicability of the signature of nine immune-relevant genes, we conducted a risk-stratified analysis based on the OS-related clinicopathological features, and we found that the signature allowed the evaluation of the immune-relevant gene risk score in subgroups by accurately assigning these variable samples to low-risk groups with longer OS and high-risk groups with shorter OS. The results demonstrated that our signature might contribute to discriminate survival outcomes of patients with GC and different clinical variables, such as age, sex, T and N stages. These findings were validated in another independent external dataset. The results from multivariate Cox analyses further confirmed that the signature of nine immune-relevant genes served as an independent risk factor for OS among patients with GC in both the test and validation cohorts.

The innate and adaptive immune systems play a crucial role in the occurrence and development of cancer [25]. In this study, we used GO and KEGG enrichment analyses to better understand the potential function of immune-relevant genes. The results showed that these immune-related genes were actively involved in the cytokine–cytokine receptor interaction, humoral immune response, and acute inflammatory response, functioning as significant parts in the inflammatory process of tumor initiation and progression [26] (Figure 5). These signaling pathways may directly or indirectly affect tumor cells in the tumor microenvironment through chronic inflammatory reactions, free radicals, and other signaling pathways [8,27]. They may inhibit the development and progression of tumors and are also verified to be effective in the treatment of cancer [28,29]. Future research works might uncover therapeutic directions for tumor immunotherapy by elucidating the mechanisms of cytokines and immune response.

However, our study had several limitations. First, the signature was developed using retrospective data. Therefore, a clinical validation of a sufficient number of GC samples is needed to prove the clinical value of this survival-predicting model. In addition, patients treated with immune checkpoint inhibitors could not confirm the association between the signature of the immune-relevant genes and the response to tumor immunotherapy.

## 5 Conclusions

In conclusion, we constructed an immune-relevant gene signature, which has a prognostic value for patients with gastric cancer and serves as an independent prognostic factor for OS among these patients. The identification of immune-relevant genes may provide new targets for research on the molecular mechanisms and personalized treatment decisions for patients with gastric cancer.
